# Longitudinal Quantiles of Frailty Trajectories Considering Death: New Insights into Sex and Cohort Differences in the Reference Curves for Frailty Progression of Older European

**DOI:** 10.1093/gerona/glae060

**Published:** 2024-02-23

**Authors:** Alejandra Marroig, Fernando Massa, Annie Robitaille, Scott M Hofer, Erwin Stolz, Graciela Muniz-Terrera

**Affiliations:** Instituto de Estadística, Universidad de la República, Montevideo, Uruguay; Instituto de Estadística, Universidad de la República, Montevideo, Uruguay; University of Ottawa, Ottawa, Ontario, Canada; Perley Health Centre of Excellence, Ottawa, Ontario, Canada.; Pacific Health Research and Education Institute, Honolulu, Hawaii; Medical University of Graz, Graz, Styria, Austria; Ohio University Heritage College of Osteopathic Medicine, Ohio University, Athens, Ohio, USA; University of Edinburgh, Edinburgh, UK; (Medical Sciences Section)

**Keywords:** Aging, Frailty, Generalized estimating equations, Mortal cohort

## Abstract

**Background:**

Most previous studies of frailty trajectories in older adults focus on the average trajectory and ignore death. Longitudinal quantile analysis of frailty trajectories permits the definition of reference curves, and the application of mortal cohort inference provides more realistic estimates than models that ignore death.

**Methods:**

Using data from individuals aged 65 or older (*n* = 25 446) from the Survey of Health, Ageing, and Retirement in Europe (SHARE) from 2004 to 2020, we derived repeated values of the Frailty Index (FI) based on the accumulation of health deficits. We applied weighted Generalized Estimating Equations to estimate the quantiles of the FI trajectory, adjusting for sample attrition due to death, sex, education, and cohort.

**Results:**

The FI quantiles increased with age and progressed faster for those with the highest level of frailty (${\hat{\beta }}_{a}^{0.9}$ = 0.0229, *p* < .001; ${\hat{\beta }}_{a}^{0.5}$ = 0.0067, *p* < .001; H_0_: $\beta_{a}^{0.5}=\beta_{a}^{0.9}$, *p* < .001). Education was consistently associated with a slower progression of the FI in all quantiles (${\hat{\beta }}_{ae}^{0.1}$ = −0.0001, *p* < .001; ${\hat{\beta }}_{ae}^{0.5}$ =−0.0004, *p* < .001; ${\hat{\beta }}_{ae}^{0.9}$ = −0.0003, *p* < .001) but sex differences varied across the quantiles. Women with the highest level of frailty showed a slower progression of the FI than men when considering death. Finally, no cohort effects were observed for the FI progression.

**Conclusions:**

Quantile FI trajectories varied by age, sex, education, and cohort. These differences could inform the practice of interventions aimed at older adults with the highest level of frailty.

Frailty, a syndrome that is characterized by individuals’ increased vulnerability to endogenous and exogenous stressors (1–3), has been reported as a key predictor of mortality, falls, hospitalizations, and other negative outcomes in older adults (4–7). Frailty is highly prevalent in older adults, especially in the older old (8), and has also been shown to be associated with increased costs for social and healthcare systems (9), imposing a burden on individuals and society (10). Thus, the identification of individuals at higher risk of frailty, and a better understanding of frailty progression and its risk factors are critical for the recommendation of interventions and the design of public health measures.

There are 2 common operationalizations of frailty, the frailty phenotype and the Frailty Index (FI) (1,3,11). The frailty phenotype is a clinical tool that is based on 5 criteria assessing the presence of symptoms and allows for the stratification of individuals into discrete states such as nonfrail, prefrail, and frail. The FI, on the other hand, is a multidimensional continuous measure that counts the number of deficits present in an individual. The FI is robust to variations in the specific deficits considered in its derivation (12) and is one of the most common frailty assessment tools (2). These complementary instruments have been recommended for use in different contexts and for different purposes (13), but because of the more fine-grained nature of the FI, most of previous studies chose this tool for the description of trajectories over time (14).

In a recent systematic review, Welstead and colleagues (14) reported inconsistencies in how frailty progresses over time due to several factors including the use of different analytical methods to model frailty progression, differences in risk factors considered, and differences in the populations studied. Furthermore, differences in the frailty tools used in the literature are also likely to majorly contribute to the inconsistencies found in the literature. In the systematic review, the authors mentioned 2 main tools used to assess frailty and, also they reported differences in how these were operationalized. For instance, the studies using the FI consider deficits ranging between 30 and 60 items.

Results about the role of age, sex, and education, 3 commonly studied sociodemographic factors, on frailty progression were also found to be inconsistent. Notably, the evidence about sex differences in frailty progression was found to be mixed (14). For instance, Stolz and colleagues (15) found that women had faster frailty progression than men, whereas Marshall and colleagues (16) did not find evidence in support of sex differences in frailty and similarly, Jenkins and colleagues (17) found no significant sex differences in frailty progression in 4 of the studies analyzed but reported that women progressed at a faster rate than men in the Health and Retirement Study (HRS).

Education was reported to be protective of frailty in some studies (17,18). However, there were inconsistencies across studies on the association of education with the level of frailty at study entry and rate of frailty progression. Jenkins and colleagues (17) reported that education was associated with lower levels of frailty at study entry in all 5 studies analyzed but associated with a slower rate of frailty progression albeit with very small effects only in 3 of the 5 studies analyzed.

There is also extensive literature showing that life expectancy has been increasing (19,20), with later-born cohorts living longer and in better health. Yet, little is known about whether cohort effects also exist in frailty trajectories. For instance, Bäckman and colleagues (21) observed stable levels of frailty comparing 2 cohorts. Yet, other studies (16,22,23) showed increasing trends in frailty for younger cohorts of older adults. Further, results regarding differences in health status between individuals born before and after World War II (24–28) are mixed. Rice and colleagues (26) reported that individuals born after 1946 in England have improved cardiovascular health, although they also have a higher prevalence of mental illness diagnoses, and do not improve their self-rated health. In contrast, a study from Lausanne reported no significant differences between older “baby boomers” and earlier cohorts and showed evidence against the compression of morbidity (27).

Frailty progression has also been shown to be a heterogeneous process (14), as various studies have identified groups of individuals with distinct frailty typologies (29,30) using mixture models. Indeed, Welstead and colleagues (31) identified 3 subgroups of individuals with distinct frailty trajectories and Verghese and colleagues (32) identified 4 subgroups of individuals with distinct frailty progression. Together, this body of literature suggests considerable individual differences in the progression of frailty and that a sole focus on the average population change provides limited insights of its progression. Further, there is a lack of knowledge about reference curves of frailty progression, which hampers the ability of clinicians and researchers to investigate the progression of frailty with a comprehensive perspective of the distribution of frailty. Moreover, our results contribute to the development of longitudinal norms (reference curves) of frailty so that clinicians and health practitioners can identify those individuals whose change in frailty deviates from the expected change for someone of similar characteristics in terms of age, sex, and education. Hence, the understanding of the distribution of frailty trajectories beyond the mean frailty progression of a population, may provide important insights into frailty change.

In addition to our narrowed understanding of frailty progression, most of the previous studies that examined change in frailty used analytical methods that ignore missing data due to death, and that results in estimates of frailty progression of immortal cohorts (33). This problem will likely be most important for the frailest individuals, as frailty is highly associated with death. Hence, to improve our understanding of frailty trajectories and generate realistic reference curves, it is critical to take attrition due to death into account.

Recent developments in quantile regression (QR) for longitudinal data in the presence of death and dropout provide the opportunity for the development of longitudinal reference curves in studies of older adults where both sources of attrition operate (34). This method constructs longitudinal quantiles of the trajectory using Generalized Estimating Equations (GEE) to account for the within-subject correlation and applies a weighted procedure under missing at random (MAR) assumption to account for attrition due to death (34).

Hence, in this study, we aim to advance knowledge about frailty progression by generating reference curves of progression of a population representative study of older adults, the Survey of Health Aging and Retirement in Europe (SHARE) considering death. More specifically, the objectives of this study are: (1) to develop reference curves for frailty using data from SHARE, a well-established population representative of European older adults, in the period 2004–2020; (2) to estimate the association of sex and education with frailty change over time for individuals at different levels of frailty trajectory; and (3) to evaluate the role of different cohorts on the FI trajectories accounting for sex and education. Our analyses consider intermittent missing data, dropout, and attrition due to death to reduce biases in the inferences, particularly when estimating the sex and cohort effects in mortal cohorts.

## Method

### Data

We used data from the SHARE, a multidisciplinary and cross-national panel database where information on health, socioeconomic status, and social and family networks of individuals aged 50 or older in Europe has been collected since 2004 across European countries (35,36).

This study uses pooled data from 9 European countries (Austria, Belgium, Denmark, France, Germany, Italy, Spain, Sweden, and Switzerland) of people aged 65 or older at study entry. Information was collected in the period 2004–2020, approximately every 2 years and corresponds to waves 1–8 of the SHARE study. Due to the special questionnaire in wave 3 and the study design in wave 7, these waves were excluded from the analysis and were used only to gather information on death of individuals. For our purposes, we included respondents for whom valid data on frailty (>99%) and years of education (>74%) at their first assessment were available, resulting in an analytical sample of *n* = 25 446 individuals with 68 845 person-wave observations.

### Frailty Index

We derived a FI based on the accumulation of deficits (12) using 40 items based on standard procedures (15,37,38). The items were selected from multiple dimensions of physiological systems: self-reported health, diagnosed morbidities, mobility, difficulties to perform basic and instrumental activities of daily living, sensory impairment, body mass index deficit, and grip strength (see the list of items and cutoff points in Supplementary Table S1). Most items showed less than 5% missing values across all times, some items showed between 11% and 15% missing values (“Engagement in activities requiring moderate level of energy”, “Reduced appetite”, “Experienced fatigue”, “Impaired vision: distance (with glasses/lenses)”, “Impaired vision: closeness (with glasses/lenses)”, “Impaired hearing”, and “BMI deficit”). The item “Deficit in Grip Strength by BMI” showed 21.8% of missing values and “Impaired orientation (date, month, year, day of week)” 32.4%. The high number of missing values in the latter is due to the fact that in waves 4 and onward only refreshment sample participants were asked the questions for “Impaired orientation". The lack of longitudinal information on this item could result in not observing a change with respect to the baseline condition. However, since the 40 items were used to calculate the FI, the overall effect is expected to be small. Nonetheless, “Deficit in Grip Strength by BMI” and “Impaired orientation” were included for their topical relevance.

The FI (range = 0–1) was computed by dividing the number of deficit items for all respondents who provided valid information in at least 35 out of 40 items by the total sum score (>99% of the respondents in each wave).

### Death

Death of participants was recorded from the end-of-life questionnaire that is carried out by the interviewers with a proxy-respondent. In the analytical sample, 22% of the participants were deceased, 27% were interviewed at wave 8, the last wave considered, and 51% were last interviewed at some wave before wave 8 but it is not known whether the person died or was not reinterviewed for another reason.

### Covariates

Education was measured using reported number of years in full-time education centered at mean (*E*). We also adjusted the FI quantiles by sex (*S*: 1 = female, 0 = male), age at follow-up (*A*) and age at study entry (*BA*) both in years and centered at 65 years old. Finally, in a separate analysis, we included an indicator of the individual’s birth cohort. We assessed the cohort effect from individuals born since 1946 (*Bo*: 1 = born in 1946 or later, 0 = born before 1946) in keeping with previous studies (25–27).

### Statistical Approach

We applied GEE to adjust the quantiles of the FI trajectory.

For individual i at occasion j, the model for the quantile τ$\left(Q^{\;\tau }(FI_{ij}|X_{ij})\right)$ was defined by:


$$\begin{array}{lll} Q^{\;\tau }(FI_{ij}|X_{ij})= & \;\beta_{0}^{\tau }+\beta_{s}^{\tau }\times S_{i}+\beta_{e}^{\tau }\times E_{i}+\beta_{ba}^{\tau }\times BA_{i}+\beta_{a}^{\tau }\times A_{ij}\; & +\beta_{as}^{\tau }\times A_{ij}\times S_{i}+\beta_{ae}^{\tau }\times A_{ij}\times E_{i}+\beta_{aba}^{\tau }\times A_{ij}\times BA_{i} \end{array}$$

This model follows a linear trajectory for the FI with age $\left(A_{ij}\right)$, with intercept $\left(\beta_{0}^{\tau }\right)$ and rate of change $\left(\beta_{a}^{\tau }\right)$ adjusted by sex $\left(S_{i}\right)$, education $\left(E_{i}\right)$, and age at study entry $\left(BA_{i}\right)$, as recommended by previous analyses of frailty trajectories (17). The changes in the intercept by variables sex, education, and baseline age are represented with the coefficients $\beta_{s}^{\tau }$, $\beta_{e}^{\tau }$, and $\beta_{ba}^{\tau }$ respectively. The changes in the slope by variables sex, education and baseline age are represented with the coefficients $\beta_{as}^{\tau }$, $\beta_{ae}^{\tau }$, and $\beta_{aba}^{\tau }$ respectively.

The model for the quantile τ$\left(Q^{\;\tau }(FI_{ij}|X_{ij})\right)$ of the FI trajectory including cohort effects ($Bo_{i}$ coded as 1 if individuals were born in 1946 or later, and 0 if individuals were born before 1946) was defined as follows:


$$\begin{array}{llll} Q^{\;\tau }\left(FI_{ij}|X_{ij}\right)= & \;\beta_{0}^{\tau }+\beta_{s}^{\tau }\times S_{i}+\beta_{e}^{\tau }\times E_{i}+\beta_{ba}^{\tau }\times BA_{i}\; & +\beta_{bo}^{\tau }\times Bo_{i}+\beta_{a}^{\tau }\times A_{ij}+\beta_{as}^{\tau }\times A_{ij}\times S_{i}+\beta_{ae}^{\tau }\; & \times A_{ij}\times E_{i}+\beta_{aba}^{\tau }\times A_{ij}\times BA_{i}+\beta_{abo}^{\tau }\times A_{ij}\times Bo_{i} \end{array}$$

In this model, the coefficient $\beta_{bo}^{\tau }\;$ represents the change in the intercept and $\beta_{abo}^{\tau }$ the change in the slope for individuals born in 1946 or later.

We estimated the marginal quantiles based on an independence working assumption using standard software for QR, specifically the *quantreg* R package (39,40). Additionally, we derived stabilized weights for the observations to account for intermittent missing data, dropout, and death, using the procedure proposed by Jacqmin-Gadda and colleagues (34). The weight estimation was developed for QR models using a GEE approach under MAR assumption. In particular, we assumed that the probability of being observed at each occasion does not depend on the survival time, which is referred to as unconditional MAR (34). Jacqmin-Gadda and colleagues (34) showed that, in mortal cohorts the weighted estimation under unconditional MAR assumption confers only a negligible bias when, in fact, the missing not at random (MNAR) is the most plausible one. We estimated the conditional probabilities of being observed at each occasion using logistic regressions in the 2nd through the last visit using the pooled samples of alive individuals. Firstly, the models were estimated under the unconditional MAR assumption, denoting the probability of being observed in the visit j as $P\left(R_{ij}=1|Al_{ij}=1,\;{\bar{X}}_{ij},{\bar{FI}}_{ik}\right)$, being $Al_{ij}$ equal to 1 if the individual i is alive at occasion j. The vector ${\bar{X}}_{ij}$ is the vector of covariates. Hence, the logistic regressions under MAR assumption were adjusted by sex, an indicator of study entry, the FI at the previous visit (if available), an indicator of been observed in the previous visit, and the interaction between FI at previous visit and sex. When the FI at previous visit was not available, the value was imputed using the mean of FI at baseline, following previous recommendations (34). Then, the models were estimated under missing completely at random (MCAR) assumption, denoting $P\left(R_{ij}=1|Al_{ij}=1,\;{\bar{X}}_{ij}\right)$ as the probability of being observed at the visit j under MCAR. Then, the logistic regressions under MCAR assumption were adjusted by sex and the indicator of study entry. Finally, the stabilized weight $\left(w_{ij}^{*}\right)$for the individual i at occasion j was obtained by dividing both conditional probabilities:


$$\begin{array}{lll} w_{ij}^{*}= & P\left(R_{ij}=1|Al_{ij}=1,\;{\bar{X}}_{ij}\right)/\; & P\left(R_{ij}=1|Al_{ij}=1,\;{\bar{X}}_{ij},{\bar{FI}}_{ik}\right)\;1\leq k<j \end{array}$$

To account for the within-subject correlation, we computed robust standard errors using a bootstrap for longitudinal data (34). We used 200 bootstrap samples to compute the standard errors of the coefficients, resampling the individuals and not the observations to preserve the correlation structure. The estimation of the weights and the standard errors were implemented using the *weightQuant* R package (34,41). Using robust standard errors, we test whether the effects of covariates were statistically significant and if there were differences in the covariate effects across the quantiles of the FI trajectory (34).

Finally, we constructed longitudinal curves of FI trajectory for women and men, using the average value of education and 65 years at study entry, accounting for both missing data and death.

## Results

### Descriptive Statistics

Table 1 shows the descriptive characteristics at study entry and the distribution of the analytical sample (*N* = 25 446) according to the number of interviews. The FI ranged from 0 to 0.886, and its mean value at baseline was 0.157 (*SD* = 0.137, 99th quantile 0.654). The median value of the FI was 0.119 and was lower than the mean, indicating that the FI follows a skewed distribution with a right tail. Almost 53% of the sample were women, with an average age of 73.8 years (*SD* = 6.8 years) at study entry, and an average of 9.6 (*SD* = 4.5 years) years of education at baseline. Over 86% (*N* = 24 364) of the individuals had less than 6 interviews completed and 21% (*N* = 5 146) of the sample died during the study period.

**Table 1.** Descriptive Statistics of Analytical Sample

Baseline	Quantiles				
	Mean	*SD*	*τ* = 0.1	*τ* = 0.5	*τ* = 0.9
All sample (*N* = 25 446)					
Frailty Index (range = 0–1)	0.157	0.137	0.031	0.119	0.344
Age (in years)	73.8	6.8	66.1	72.5	83.6
Education (in years)	9.6	4.5	4	9	16
Sex	*N*	%			
Women	13 427	52.77			
Men	12 019	47.23			
Born before 1946 (*N* = 23 337)					
Frailty Index (range = 0–1)	0.161	0.139	0.031	0.119	0.350
Age (in years)	74.5	6.6	66.6	73.3	84.0
Education (in years)	9.4	4.5	4	9	15
Sex	*N*	%			
Women	12 388	53.08			
Men	10 949	46.92			
Born 1946 or after (*N* = 2 109)					
Frailty Index (range 0–1)	0.111	0.101	0.019	0.087	0.229
Age (in years)	66.4	1.17	65.2	66.2	67.7
Education (in years)	11.1	4.6	5	11	17
Sex	*N*	%			
Women	1 039	49.27			
Men	1 070	50.73			
Interviews	*N*	%			
Six	1 082	4.25			
Five	2 171	8.53			
Four	3 351	13.17			
Three	5 983	23.51			
Two	7 286	28.63			
One	5 573	21.90			

*Note*: *SD =* standard deviation.

### Longitudinal Quantiles of Frailty Trajectory and Their Association With Sex and Education

Table 2 shows the results of the QR for the FI trajectory using weighted and unweighted procedures, both using robust standard errors. The results showed that the median FI increases with age in both the weighted (${\hat{\beta }}_{a}^{0.5}$ = 0.0067, *p* < .001) and the unweighted procedure (${\hat{\beta }}_{a}^{0.5UW}$ = 0.0031, *p* < .001). Furthermore, education significantly reduces the median FI increase with age in both procedures (${\hat{\beta }}_{ae}^{0.5}$ = −0.0004, *p* < .001 weighted and ${\hat{\beta }}_{ae}^{0.5UW}$ = −0.0002, *p* < .001 unweighted).

**Table 2.** Estimates of the Quantile Regressions. Outcome Variable FI (Range = 0–1)

		${\hat{\beta }}_{v}^{0.1}$ (*SE*)	${\hat{\beta }}_{v}^{0.5}$ (*SE*)	${\hat{\beta }}_{v}^{0.9}$ (*SE*)	τ = 0.1 vs 0.5 *p* value^†^	τ = 0.5 vs 0.9 *p* value^‡^
Quantile regression weighted—intermittent missing data and death						
Sex	${\hat{\beta }}_{s}^{\tau }$	−0.0062^*^ (0.0016)	0.0036 (0.0028)	0.0174^*^ (0.0084)	<.001	.076
Educ	${\hat{\beta }}_{e}^{\tau }$	−0.0004^*^ (0.0002)	−0.0008^*^ (0.0003)	−0.0082^*^ (0.0009)	.190	<.001
BAge	${\hat{\beta }}_{ba}^{\tau }$	−0.0027^*^ (0.0003)	−0.0070^*^ (0.0005)	−0.0053^*^ (0.0014)	<.001	.183
Age	${\hat{\beta }}_{a}^{\tau }$	0.0022^*^ (0.0002)	0.0067^*^ (0.0004)	0.0229^*^ (0.0010)	<.001	<.001
Age.Sex	${\hat{\beta }}_{as}^{\tau }$	0.0011^*^ (0.0002)	0.0020^*^ (0.0004)	−0.0013^*^ (0.0006)	.010	<.001
Age.Educ	${\hat{\beta }}_{ae}^{\tau }$	−0.0001^*^ (0.00002)	−0.0004^*^ (0.00004)	−0.0003^*^ (0.0001)	<.001	.013
Age.BAge	${\hat{\beta }}_{aba}^{\tau }$	0.0001^*^ (0.00001)	0.0004^*^ (0.00002)	−0.00004 (0.00004)	<.001	<.001
Quantile regression unweighted						
Sex	${\hat{\beta }}_{s}^{\tau UW}$	−0.0071^*^ (0.0014)	0.0010 (0.0021)	0.0354^*^ (0.0053)	<.001	<.001
Edu	${\hat{\beta }}_{e}^{\tau UW}$	−0.0007^*^ (0.0002)	−0.0014^*^ (0.0003)	−0.0062^*^ (0.0005)	.002	<.001
BAge	${\hat{\beta }}_{ba}^{\tau UW}$	−0.0012^*^ (0.0002)	−0.0034^*^ (0.0003)	−0.0030^*^ (0.0008)	<.001	.618
Age	${\hat{\beta }}_{a}^{\tau UW}$	0.0010^*^ (0.0002)	0.0031^*^ (0.0002)	0.0115^*^ (0.0005)	<.001	<.001
Age.Sex	${\hat{\beta }}_{as}^{\tau UW}$	0.0012^*^ (0.0001)	0.0024^*^ (0.0002)	0.0008 (0.0005)	<.001	<.001
Age.Educ	${\hat{\beta }}_{ae}^{\tau UW}$	−0.0001^*^ (0.00002)	−0.0002^*^ (0.00004)	−0.0003^*^ (0.00004)	<.001	.071
Age.BAge	${\hat{\beta }}_{aba}^{\tau UW}$	0.0001^*^ (0.00001)	0.0003^*^ (0.00002)	0.0002^*^ (0.00003)	<.001	.270

*Notes*: Robust standard errors in parenthesis. Age centered at 65 years old. Educ = education (in years centered at mean of 9.6 years); BAge = baseline age (in years centered at 65 years old). Boom: indicator, 1 = born in 1946 or later, 0 = born before 1946. Interactions of variables are symbolized with a dot.

^*^ *p* < .05 for H_0_: $\beta_{v}^{\tau }=0$.

^†^ *p* Value for H_0_: $\beta_{v}^{0.1}=\beta_{v}^{0.5}$.

^‡^ *p* Value for H_0_: $\beta_{v}^{0.5}=\beta_{v}^{0.9}$.

In addition, the median FI at 65 years of age did not differ significantly between men and women (${\hat{\beta }}_{s}^{0.5}$ = 0.0036, *p* = .194 weighted and ${\hat{\beta }}_{s}^{0.5UW}$ = 0.0010, *p* = .648 unweighted). However, the progression with age of the median FI was higher for women than for men (${\hat{\beta }}_{as}^{0.5}$ = 0.0020, *p* < .001 weighted and ${\hat{\beta }}_{as}^{0.5UW}$ = 0.0024, *p* < .001 unweighted procedure). Finally, a steeper increase with age of the median FI was obtained for those entering the study at older ages (${\hat{\beta }}_{aba}^{0.5}$ = 0.0004, *p* < .001; ${\hat{\beta }}_{aba}^{0.5UW}$ = 0.0003, *p* < .001).

The FI trajectory differs across quantiles. For those who are more vulnerable (quantile τ = 0.9), the progression of the FI is steeper than for those at the median (${\hat{\beta }}_{a}^{0.9}$ = 0.0229, *p* < .001; ${\hat{\beta }}_{a}^{0.5}$ = 0.0067, *p* < .001; test H_0_: $\beta_{a}^{0.5}=\beta_{a}^{0.9}$, *p* < .001). This result was also obtained for the unweighted procedure (${\hat{\beta }}_{a}^{0.9UW}$ = 0.0115, *p* < .001; ${\hat{\beta }}_{a}^{0.5UW}$ = 0.0031, *p* < .001; test H_0_: $\beta_{a}^{0.5UW}=\beta_{a}^{0.9UW}$, *p* < .001). In contrast, FI progression is slower for the least frail (quantile τ = 0.1) than for those at the median (${\hat{\beta }}_{a}^{0.1}$ = 0.0022, *p* < .001; ${\hat{\beta }}_{a}^{0.5}$ = 0.0067, *p* < .001; test H_0_: $\beta_{a}^{0.1}=\beta_{a}^{0.5}$, *p* < .001).

Furthermore, the weighted and unweighted procedures were inconsistent for the most frail (quantile τ = 0.9) but not for the median nor for the least frail (quantile τ = 0.1) of the FI trajectory. In fact, unlike the progression of the median and the quantile τ = 0.1, the increase with age of the FI quantile τ = 0.9 is slower for women than for men in the weighted procedure that considers missing data and death (${\hat{\beta }}_{as}^{0.9}$ = −0.0013, *p* = .032).

As previously mentioned for the median, consistently across the quantiles (τ = 0.1 and 0.9) a higher level of education is associated with a reduced progression of the FI (${\hat{\beta }}_{ae}^{0.1}$ = −0.0001, *p* < .001; ${\hat{\beta }}_{ae}^{0.5}$ = −0.0004, *p* < .001; ${\hat{\beta }}_{ae}^{0.5}$ = −0.0003, *p* < .001). However, the magnitude of the reductions differs across quantiles (test H_0_: $\beta_{ae}^{0.1}=\beta_{ae}^{0.5}$, *p* < .001; test H_0_: $\beta_{ae}^{0.5}=\beta_{ae}^{0.9}$, *p* = .013). It follows that the biggest reduction in FI progression with age resulting from increasing education corresponds to the median trajectory, then to the quantile τ = 0.9 and being the smallest for the quantile τ = 0.1.

**Figure 1.**
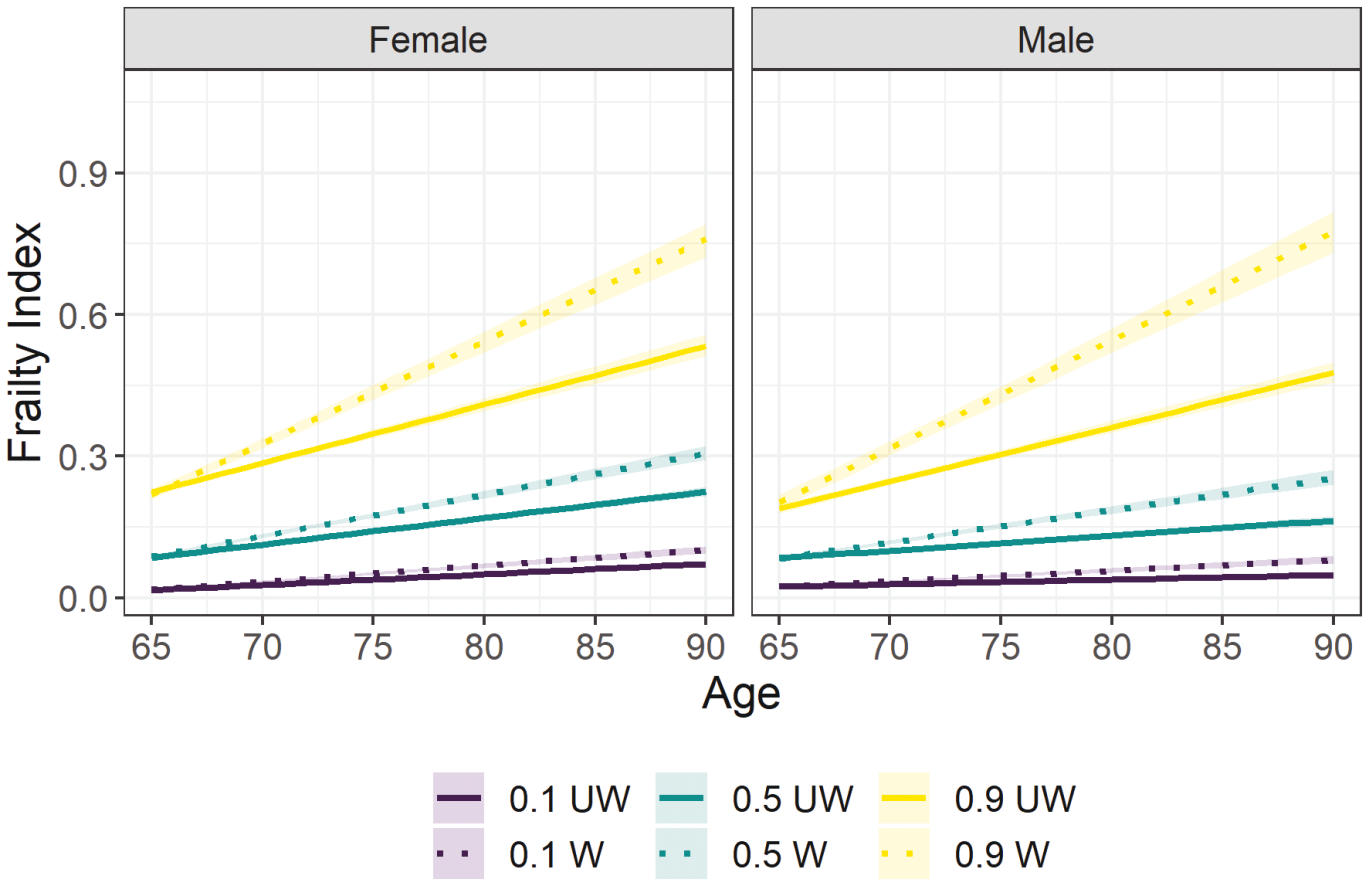
Adjusted FI trajectories for quantiles τ = 0.1, 0.5 (median), 0.9. Notes: Trajectory for quantiles 𝜏 = 0.1, 0.5, and 0.9 of the FI (range 0–1). W = weighted predicted FI trajectories with intermittent missing data and death; UW = unweighted predicted FI trajectories. Education at baseline mean. 95% confident intervals of the FI trajectories.

Figure 1 depicts the predicted FI trajectories for women and men. The trajectories were obtained for mean education, considering missing data and death (weighted procedure) and not (unweighted procedure). The results showed that the weighted trajectory has a steeper progression of the FI with the same point of departure at 65 years old for women and men. In addition, we observed a steeper progression for the quantile τ = 0.9 than for the median or the quantile τ = 0.1 and a similar pattern was found for men and women.

Women living with the highest level of frailty (quantile τ = 0.9) had a slower progression of FI than men using the weighted procedure. However, this was not evident when using the unweighted analysis. The progression of FI for individuals with the highest level of frailty is slower for women than for men (${\hat{\beta }}_{as}^{0.9}$ = −0.013, *p* = .032) considering death, but there were no differences using the unweighted procedure (${\hat{\beta }}_{as}^{0.9UW}$ = 0.0008, *p* = .078). Furthermore, the progression of the median is steeper for men than women for both procedures (${\hat{\beta }}_{as}^{0.5}$ = 0.0020, *p* < .001 and ${\hat{\beta }}_{as}^{0.5UW}$ = 0.0024, *p* < .001). Finally, the results showed higher levels of the reference curves (longitudinal quantiles) of the median and the quantile τ = 0.1 for women than for men, and this result was more pronounced when using the weighted procedure (see Figure 1).

### Longitudinal Quantiles of Frailty Trajectory and Their Association With Sex and Education in Different Cohorts

The results for weighted and unweighted analysis including cohort effects showed robustness regarding the effects of age, sex, education, and baseline age on the quantiles of the FI trajectory (see Table 3). All the FI quantile trajectories increased with age and the increases were different across the quantiles. The weighted analysis, which introduces the intermittent missing data process and death in the inferences, showed different results only for the individuals with higher levels of frailty (quantile τ = 0.9). Higher education significantly reduced the progression of the FI consistently across the quantiles (Table 3).

**Table 3.** Estimates of the Quantile Regressions With Cohort Effects. Outcome Variable FI (Range = 0–1)

		${\hat{\beta }}_{v}^{0.1}$ (*SE*)	${\hat{\beta }}_{v}^{0.5}$ (*SE*)	${\hat{\beta }}_{v}^{0.9}$ (*SE*)	τ = 0.1 vs 0.5 *p* value^†^	τ = 0.5 vs 0.9 *p* value^‡^
Quantile regression weighted—intermittent missing data and death						
Sex	${\hat{\beta }}_{s}^{\tau }$	−0.0064^*^ (0.0015)	0.0037 (0.0028)	0.0244^*^ (0.0085)	<.001	<.001
Educ	${\hat{\beta }}_{e}^{\tau }$	−0.0005^*^ (0.0002)	−0.0008^*^ (0.0003)	−0.0085^*^ (0.0008)	.219	<.001
BAge	${\hat{\beta }}_{ba}^{\tau }$	−0.0027^*^ (0.0003)	−0.0067^*^ (0.0005)	−0.0036^*^ (0.0014)	<.001	.014
Boom	${\hat{\beta }}_{bo}^{\tau }$	0.0019 (0.0019)	0.0080^*^ (0.0034)	0.0690^*^ (0.0162)	.060	<.001
Age	${\hat{\beta }}_{a}^{\tau }$	0.0023^*^ (0.0002)	0.0070^*^ (0.0004)	0.0224^*^ (0.0010)	<.001	<.001
Age.Sex	${\hat{\beta }}_{as}^{\tau }$	0.0011^*^ (0.0002)	0.0019^*^ (0.0004)	−0.0018^*^ (0.0006)	.012	<.001
Age.Educ	${\hat{\beta }}_{ae}^{\tau }$	−0.0001^*^ (0.0001)	−0.0004^*^ (0.0001)	−0.0003^*^ (0.0001)	<.001	.003
Age.BAge	${\hat{\beta }}_{aba}^{\tau }$	0.0001^*^ (0.0001)	0.0003^*^ (0.0001)	−0.0004^*^ (0.0001)	<.001	<.001
Age.Boom	${\hat{\beta }}_{abo}^{\tau }$	0.0002 (0.0007)	0.0007 (0.0013)	0.0033 (0.0078)	.711	.724
Quantile regression unweighted						
Sex	${\hat{\beta }}_{s}^{\tau UW}$	−0.0072^*^ (0.0013)	0.7021^*^ (0.0021)	0.0348^*^ (0.0056)	<.001	<.001
Educ	${\hat{\beta }}_{e}^{\tau UW}$	−0.0007^*^ (0.0002)	0.0001^*^ (0.0003)	−0.0063^*^ (0.0005)	.003	<.001
BAge	${\hat{\beta }}_{ba}^{\tau UW}$	−0.0012^*^ (0.0003)	0.0001^*^ (0.0004)	−0.0026^*^ (0.0009)	<.001	.245
Boom	${\hat{\beta }}_{bo}^{\tau UW}$	0.0018 (0.0017)	0.2514^*^ (0.0027)	0.0302^*^ (0.0093)	.612	.001
Age	${\hat{\beta }}_{a}^{\tau UW}$	0.0010^*^ (0.0002)	0.0001^*^ (0.0002)	0.0120^*^ (0.0005)	<.001	<.001
Age.Sex	${\hat{\beta }}_{as}^{\tau UW}$	0.0012^*^ (0.0001)	0.0001^*^ (0.0002)	0.0009 (0.0005)	<.001	<.001
Age.Educ	${\hat{\beta }}_{ae}^{\tau UW}$	−0.0001^*^ (0.0001)	0.0001^*^ (0.0001)	−0.0003^*^ (0.0001)	<.001	.158
Age.BAge	${\hat{\beta }}_{aba}^{\tau UW}$	0.0001^*^ (0.0001)	0.0001^*^ (0.0001)	0.0002^*^ (0.0001)	<.001	.034
Age.Boom	${\hat{\beta }}_{abo}^{\tau UW}$	−0.0015^*^ (0.0005)	0.0013^*^ (0.0005)	−0.0060^*^ (0.0018)	.684	.010

*Notes*: Robust standard errors in parenthesis. Age centered at 65 years old. Educ = education (in years centered at mean of 9.6 years); BAge = baseline age (in years centered at 65 years old). Boom: indicator, 1 = born in 1946 or later, 0 = born before 1946. Interactions of variables are symbolized with a dot.

^*^ *p* < .05 for H_0_: $\beta_{v}^{\tau }=0$.

^†^ *p* Value for H_0_: $\beta_{v}^{0.1}=\beta_{v}^{0.5}$_._

^‡^ *p* Value for H_0_: $\beta_{v}^{0.5}=\beta_{v}^{0.9}$.

In addition, the results showed that being born in 1946 or later was associated with a higher level of FI at baseline for the median (${\hat{\beta }}_{bo}^{0.5}$ = 0.0080, *p* = .020) and the quantile τ = 0.9 (${\hat{\beta }}_{bo}^{0.9}$ = 0.0690 *p* < .001), but no significant differences were observed for the quantile τ = 0.1 (${\hat{\beta }}_{bo}^{0.1}$ = 0.0019, *p* = .317).

Being born in 1946 or later was associated with a significant reduction of the progression with age of the FI in the unweighted procedure for all the quantiles (${\hat{\beta }}_{abo}^{0.1UW}$ = −0.0015, *p* = .002, ${\hat{\beta }}_{abo}^{0.5UW}$ = −0.0017, *p* = .001, and ${\hat{\beta }}_{abo}^{0.9UW}$ = −0.0060, *p* = .001). However, these effects were not statistically significant in the weighted procedure for any of the quantiles (${\hat{\beta }}_{abo}^{0.1}$ = 0.0002, *p* = .794, ${\hat{\beta }}_{abo}^{0.5}$ = 0.0007, *p* = .621, and ${\hat{\beta }}_{abo}^{0.9}$ = 0.0033, *p* = .674).

### Sensitivity Analysis

The analyses were performed considering education for the estimation of weights to consider intermittent missing data and death. Hence, the logistic regressions under MAR assumption were adjusted by sex, education, an indicator of study entry, the FI at the previous visit (if available), an indicator of been observed in the previous visit, and the interaction between FI at previous visit with sex and with education. The main results remained unchanged (see Supplementary Table S2).

## Discussion

We assessed the heterogeneity of frailty trajectories in 9 European countries from SHARE in 2004–2020. This heterogeneity was assessed using QR to adjust the quantiles of the FI trajectory (longitudinal quantiles), considering within-subject correlation with a GEE approach. In particular, we adjusted the trajectory of the quantile τ = 0.5, which reflects the median trajectory, the quantile τ = 0.9, which reflects the evolution of the individuals with highest levels of frailty, and the quantile τ = 0.1 to consider the evolution of the individuals with lowest levels of frailty. This allowed us to understand the consistency or differences in the role of covariates (age, sex, and education) on the progression of frailty across different levels of its trajectory. In addition, we introduce a weighting procedure to account for the missing data process and death, which are often present in longitudinal studies of aging. We also analyze cohort effects in the quantiles of the FI trajectory, introducing the effect of being born in 1946 or later.

The results showed that the quantiles τ = 0.1, 0.5 (median), and 0.9 of FI increased with age and there were differences in the role of covariates in each quantile trajectory. For instance, an increase in years of education is associated with a decrease in the progression of the FI for all the quantiles, but this effect varies across the quantiles. Although this decrease is significant, it is of relatively low magnitude. Specifically, the results for the median trajectory showed that to compensate the increase in FI due to one more year of age (${\hat{\beta }}_{a}^{0.5}$ = 0.0067) would require education to increase by almost 17 years (${\hat{\beta }}_{ae}^{0.5}$ = −0.0004; ${\hat{\beta }}_{a}^{0.5}/{\hat{\beta }}_{ae}^{0.5}$ = 16.75 years).

Previous studies stated that biomedical, behavioral, or mental factors, account for educational differences in frailty, showing that lower education is associated with higher levels of frailty (42). Also, higher educational attainment could represent a socioeconomic advantage associated with lower levels of frailty (14,17). Our results add to previous findings by suggesting that lower levels of education are more relevant for those at the median, as the effect of education is significantly higher than for those with the lowest (quantile τ = 0.1) or highest (quantile τ = 0.9) levels of frailty.

Women and men showed different progressions of FI quantiles providing partial support to the male–female-health-survival paradox (43). Women have steeper progression of the median and quantile τ = 0.1 FI than men. However, women with highest level of frailty (quantile τ = 0.9) have slower frailty progression than men. This result is in keeping with previous findings that reported a higher slope of frailty mean trajectory for women compared to men in 4 cohorts (17) and a steeper progression of frailty for men in terminal decline participants of the HRS (44). Additionally, our results suggest that death may introduce biases in the estimation of sex differences in frailty progression. Individuals with the highest levels of frailty (quantile τ = 0.9) did not show sex differences when death was ignored, whereas weighted reference curves showed slower progression for women than for men. This result is consistent with a survival effect of women, who live longer with higher levels of frailty than men but not with a more pronounced progression of frailty.

It is also important to account for the missing data process and death when analyzing cohort effects. The progression with age of the FI showed the same pattern for both cohorts when we accounted for missing data and death. We think that the statistically significant reduction of the progression with age for the younger cohort obtained with the unweighted procedure may be due to a survivor effect.

Also, our results showed that the progression of the FI is steeper for those who are more vulnerable (quantile τ = 0.9) than for those at the median, in keeping with previous findings using data from SHARE (45). Some previous studies reported small improvements in frailty over time for some participants (46–48) and the authors explained these improvements by different attrition rates of individuals living with higher levels of frailty at follow-up, potentially resulting in a healthy survivor effect. In our analyses, an improving FI effect for the younger cohort was nonsignificant when considering death. This may explain the findings of previous studies as they had not considered the survivor effects in their inferences.

Although the overall findings were in keeping with previous studies, we have provided evidence on the heterogeneity of frailty trajectories and how the survival effect may affect the inferences. The role of sex, education, and age at study entry varied across quantiles implying that depending on the level of frailty the interventions may be oriented to groups with different characteristics. For instance, interventions targeted at those with the highest level of frailty (quantile τ = 0.9) may be homogeneous in terms of sex and education because the evidence showed that at that level of frailty there are no differences regarding these factors.

This article contributes to the existing literature (49,50) on the heterogeneity of frailty trajectories by estimating reference curves that account for the longitudinal nature of frailty, showing heterogeneity in the association of sex and education for different levels of frailty, and considering the missing data and death processes to reduce bias in inferences. Furthermore, our results supplement recent work on the consistency and reliability of the FI to target policies among groups of community-dwelling older adults (51). More specifically, we provide new insights into how risk factor associations vary across the longitudinal quantiles of the FI.

Our analyses have several strengths and limitations. First, estimating the quantiles of FI trajectory gives us a better understanding of the entire distribution of the FI trajectory and allows us to find differences in the role of covariates for different levels of frailty. Second, accounting for missing data processes and deaths showed differences in the results of the FI trajectory and our methodological approach accounted for these differences. This poses a new challenge for those analyzing trajectories in aging studies, where missing data and death have consistently been a challenge to incorporate into inferences. However, our results are not without limitations. More exhaustive analyses of the weighting procedure are needed which may include different variables to estimate the weights and different assumptions of the missing data process (34). Further research on weighting should be conducted to account for intermittent missing data and death. Vital status from SHARE data was reported to be unknown in considerable cases. Further research should rely on more complete mortality-follow-up data to assess the heterogeneity in frailty trajectories. In addition, understanding frailty trajectories needs to be done in different contexts with coordinated analyses showing translational consideration of the findings (17). Finally, other relevant factors, such as geographic location or lifestyle factors, may be included to achieve a broader understanding of frailty trajectories (14).

Our results highlight the need to incorporate an approach that allows to better understand frailty trajectories for older people with different levels of vulnerability. In addition, it is essential to incorporate statistical methodologies that consider death of older adults, especially for the most vulnerable groups with the highest mortality rates.

In our study, we hand-picked the quantiles τ = 0.1, τ = 0.5 (median), and τ = 0.9 which refer to low, medium, and high levels of frailty trajectories and are often used in QR, although it is possible to choose any other quantile of interest. A better understanding of the heterogeneity in frailty trajectories can improve our knowledge about individuals who are most vulnerable, which may have implications for the development of recommendations and the design of health interventions.

## Supplementary Material

glae060_suppl_Supplementary_Tables_S1-S2

## References

[1] Clegg A , Young J, Iliffe S, Rikkert MO, Rockwood K. Frailty in elderly people. Lancet (London, England). 2013;381(9868):752–762. 10.1016/S0140-6736(12)62167-9 23395245 PMC4098658

[2] Dent E , Kowal P, Hoogendijk EO. Frailty measurement in research and clinical practice: a review. Eur J Intern Med. 2016;31:3–10. 10.1016/j.ejim.2016.03.007 27039014

[3] Hoogendijk EO , Afilalo J, Ensrud KE, Kowal P, Onder G, Fried LP. Frailty: implications for clinical practice and public health. Lancet (London, England). 2019;394(10206):1365–1375. 10.1016/S0140-6736(19)31786-6 31609228

[4] Kojima G , Iliffe S, Walters K. Frailty index as a predictor of mortality: a systematic review and meta-analysis. Age Ageing. 2018;47(2):193–200. 10.1093/ageing/afx162 29040347

[5] Kojima G. Frailty as a predictor of future falls among community-dwelling older people: a systematic review and meta-analysis. J Am Med Dir Assoc. 2015;16(12):1027–1033. 10.1016/j.jamda.2015.06.018 26255098

[6] Kojima G. Frailty as a predictor of hospitalisation among community-dwelling older people: a systematic review and meta-analysis. J Epidemiol Community Health. 2016;70(7):722–729. 10.1136/jech-2015-206978 26933121

[7] Kojima G. Frailty as a predictor of disabilities among community-dwelling older people: a systematic review and meta-analysis. Disabil Rehabil. 2017;39(19):1897–1908. 10.1080/09638288.2016.1212282 27558741

[8] Manfredi G , Midão L, Paúl C, Cena C, Duarte M, Costa E. Prevalence of frailty status among the European elderly population: findings from the Survey of Health, Aging and Retirement in Europe. Geriatr Gerontol Int. 2019;19(8):723–729. 10.1111/ggi.13689 31146300

[9] Ensrud KE , Kats AM, Schousboe JT, et al.; Study of Osteoporotic Fractures. Frailty phenotype and healthcare costs and utilization in older women. J Am Geriatr Soc. 2018;66(7):1276–1283. 10.1111/jgs.15381 29684237 PMC6097947

[10] Turner G , Clegg A; British Geriatrics Society. Best practice guidelines for the management of frailty: a British Geriatrics Society, Age UK and Royal College of General Practitioners report. Age Ageing. 2014;43(6):744–747. 10.1093/ageing/afu138 25336440

[11] Fried LP , Tangen CM, Walston J, et al.; Cardiovascular Health Study Collaborative Research Group. Frailty in older adults: evidence for a phenotype. J Gerontol A Biol Sci Med Sci. 2001;56(3):M146–M156. 10.1093/gerona/56.3.m146 11253156

[12] Rockwood K , Mitnitski A. Frailty in relation to the accumulation of deficits. J Gerontol A Biol Sci Med Sci. 2007;62(7):722–727. 10.1093/gerona/62.7.722 17634318

[13] Cesari M , Gambassi G, Abellan van Kan G, Vellas B. The frailty phenotype and the frailty index: different instruments for different purposes. Age Ageing. 2014;43(1):10–12. 10.1093/ageing/aft160 24132852

[14] Welstead M , Jenkins ND, Russ T, Luciano M, Muniz-Terrera G. A systematic review of frailty trajectories: their shape and influencing factors. Gerontologist. 2021;61(8):e463–e475. 10.1093/geront/gnaa061 32485739 PMC8599181

[15] Stolz E , Mayerl H, Waxenegger A, Rásky E, Freidl W. Impact of socioeconomic position on frailty trajectories in 10 European countries: evidence from the Survey of Health, Ageing and Retirement in Europe (2004–2013). J Epidemiol Community Health. 2017;71(1):73–80. 10.1136/jech-2016-207712 27422980

[16] Marshall A , Nazroo J, Tampubolon G, Vanhoutte B. Cohort differences in the levels and trajectories of frailty among older people in England. J Epidemiol Community Health. 2015;69(4):316–321. 10.1136/jech-2014-204655 25646207 PMC4392235

[17] Jenkins ND , Hoogendijk EO, Armstrong JJ, et al. Trajectories of frailty with aging: coordinated analysis of five Longitudinal Studies. Innov Aging. 2022;6(2):igab059. 10.1093/geroni/igab059 35233470 PMC8882228

[18] Peek MK , Howrey BT, Ternent RS, Ray LA, Ottenbacher KJ. Social support, stressors, and frailty among older Mexican American adults. J Gerontol B Psychol Sci Soc Sci. 2012;67(6):755–764. 10.1093/geronb/gbs081 23009957 PMC3478725

[19] Christensen K , Doblhammer G, Rau R, Vaupel JW. Ageing populations: the challenges ahead. Lancet (London, England). 2009;374(9696):1196–1208. 10.1016/S0140-6736(09)61460-4 19801098 PMC2810516

[20] Crimmins EM , Beltrán-Sánchez H. Mortality and morbidity trends: is there compression of morbidity? J Gerontol B Psychol Sci Soc Sci. 2011;66B(1):75–86. 10.1093/geronb/gbq088 PMC3001754 21135070

[21] Bäckman K , Joas E, Falk H, Mitnitski A, Rockwood K, Skoog I. Changes in the lethality of frailty over 30 years: evidence from two cohorts of 70-year-olds in Gothenburg Sweden. J Gerontol A Biol Sci Med Sci. 2017;72(7):945–950. 10.1093/gerona/glw160 27522060 PMC5458400

[22] Yu R , Wong M, Chong KC, et al. Trajectories of frailty among Chinese older people in Hong Kong between 2001 and 2012: an age-period-cohort analysis. Age Ageing. 2018;47(2):254–261. 10.1093/ageing/afx170 29161361

[23] Hoogendijk EO , Stolz E, Oude Voshaar RC, Deeg DJH, Huisman M, Jeuring HW. Trends in frailty and its association with mortality: results from the Longitudinal Aging Study Amsterdam, 1995–2016. Am J Epidemiol. 2021;190(7):1316–1323. 10.1093/aje/kwab018 33534876 PMC8245891

[24] Martin LG , Schoeni RF, Freedman VA, Andreski P. Feeling better? Trends in general health status. J Gerontol B Psychol Sci Soc Sci. 2007;62(1):S11–S21. 10.1093/geronb/62.1.s11 17284560

[25] Badley EM , Canizares M, Perruccio AV, Hogg-Johnson S, Gignac MA. Benefits gained, benefits lost: comparing baby boomers to other generations in a longitudinal cohort study of self-rated health. Milbank Q. 2015;93(1):40–72. 10.1111/1468-0009.12105 25752350 PMC4364431

[26] Rice NE , Lang IA, Henley W, Melzer D. Baby boomers nearing retirement: the healthiest generation? Rejuvenation Res. 2010;13(1):105–114. 10.1089/rej.2009.0896 20230284

[27] Henchoz Y , Gunten A von, Büla C, et al. Do baby boomers feel healthier than earlier cohorts after retirement age? The Lausanne cohort Lc65+ study. BMJ Open. 2019;9(2):e025175. 10.1136/bmjopen-2018-025175 PMC6368217 30782927

[28] Lanzieri G. Population and social conditions. Eurostat; 2011. https://ec.europa.eu/eurostat/web/products-statistics-in-focus/-/KS-SF-11-023

[29] Stephan AJ , Strobl R, Schwettmann L, et al. The times we are born into and our lifestyle choices determine our health trajectories in older age—results from the KORA-Age study. Prev Med. 2020;133:106025. 10.1016/j.ypmed.2020.106025 32061683

[30] Stow D , Matthews FE, Hanratty B. Frailty trajectories to identify end of life: a longitudinal population-based study. BMC Med. 2018;16(1):171. 10.1186/s12916-018-1148-x 30236103 PMC6148780

[31] Welstead M , Luciano M, Russ TC, Muniz-Terrera G. Heterogeneity of frailty trajectories and associated factors in the Lothian Birth Cohort 1936. Gerontology. 2022;68(8):861–868. 10.1159/000519240 34587617 PMC9501780

[32] Verghese J , Ayers E, Sathyan S, et al. Trajectories of frailty in aging: prospective cohort study. PLoS One. 2021;16(7):e0253976. 10.1371/journal.pone.0253976 34252094 PMC8274857

[33] Rouanet A , Helmer C, Dartigues JF, Jacqmin-Gadda H. Interpretation of mixed models and marginal models with cohort attrition due to death and drop-out. Stat Methods Med Res. Published online August 8, 2017;28:343–356. 10.1177/0962280217723675 28784010

[34] Jacqmin-Gadda H , Rouanet A, Mba RD, Philipps V, Dartigues JF. Quantile regression for incomplete longitudinal data with selection by death. Stat Methods Med Res. 2020;29(9):2697–2716. 10.1177/0962280220909986 32180497

[35] Börsch-Supan A , Brandt M, Hunkler C, et al.; SHARE Central Coordination Team. Data resource profile: the Survey of Health, Ageing and Retirement in Europe (SHARE). Int J Epidemiol. 2013;42(4):992–1001. 10.1093/ije/dyt088 23778574 PMC3780997

[36] Bergmann M , Kneip T, De Luca G, Scherpenzeel A. Survey participation in the Survey of Health, Ageing and Retirement in Europe (SHARE), wave 1-7. Based on Release 7.0.0. Published online 2019. Accessed November 23, 2022. http://www.share-project.org/uploads/tx_sharepublications/WP_Series_41_2019_Bergmann_et_al.pdf

[37] Romero-Ortuno R , Kenny RA. The frailty index in Europeans: association with age and mortality. Age Ageing. 2012;41(5):684–689. 10.1093/ageing/afs051 22522775 PMC3424051

[38] Searle SD , Mitnitski A, Gahbauer EA, Gill TM, Rockwood K. A standard procedure for creating a frailty index. BMC Geriatr. 2008;8:24. 10.1186/1471-2318-8-24 18826625 PMC2573877

[39] Koenker R. Quantile regression. Cambridge University Press; 2005. 10.1017/CBO9780511754098

[40] Koenker R. Package “quantreg.” Published online July 20, 2022. Accessed September 30, 2022. https://cran.r-project.org/web/packages/quantreg/quantreg.pdf

[41] Philipps V. Package “weightQuant.” Published online January 5, 2022. Accessed September 30, 2022. https://cran.r-project.org/web/packages/weightQuant/weightQuant.pdf

[42] Hoogendijk EO , van Hout HPJ, Heymans MW, et al. Explaining the association between educational level and frailty in older adults: results from a 13-year longitudinal study in the Netherlands. Ann Epidemiol. 2014;24(7):538–44.e2. 10.1016/j.annepidem.2014.05.002 24935466

[43] Gordon EH , Peel NM, Samanta M, Theou O, Howlett SE, Hubbard RE. Sex differences in frailty: a systematic review and meta-analysis. Exp Gerontol. 2017;89:30–40. 10.1016/j.exger.2016.12.021 28043934

[44] Stolz E , Mayerl H, Hoogendijk EO, Armstrong JJ, Roller-Wirnsberger R, Freidl W. Acceleration of health deficit accumulation in late-life: evidence of terminal decline in frailty index three years before death in the US Health and Retirement Study. Ann Epidemiol. 2021;58:156–161. 10.1016/j.annepidem.2021.03.008 33812966

[45] Stolz E , Hoogendijk EO, Mayerl H, Freidl W. Frailty changes predict mortality in 4 Longitudinal Studies of Aging. J Gerontol A Biol Sci Med Sci. 2021;76(9):1619–1626. 10.1093/gerona/glaa266 33103718 PMC8361367

[46] Gajic-Veljanoski O , Papaioannou A, Kennedy C, et al.; CaMos Research Group. Osteoporotic fractures and obesity affect frailty progression: a longitudinal analysis of the Canadian multicentre osteoporosis study. BMC Geriatr. 2018;18(1):4. 10.1186/s12877-017-0692-0 29304836 PMC5756402

[47] Mitnitski A , Song X, Rockwood K. Trajectories of changes over twelve years in the health status of Canadians from late middle age. Exp Gerontol. 2012;47(12):893–899. 10.1016/j.exger.2012.06.015 22790020

[48] Rogers NT , Marshall A, Roberts CH, Demakakos P, Steptoe A, Scholes S. Physical activity and trajectories of frailty among older adults: evidence from the English Longitudinal Study of Ageing. PLoS One. 2017;12(2):e0170878. 10.1371/journal.pone.0170878 28152084 PMC5289530

[49] Pérez-Zepeda MU , Godin J, Armstrong JJ, et al. Frailty among middle-aged and older Canadians: population norms for the frailty index using the Canadian Longitudinal Study on Aging. Age Ageing. 2021;50(2):447–456. 10.1093/ageing/afaa144 32805022

[50] Sundarakumar JS , Raviteja KV, Muniz‐Terrera G, Ravindranath V. Normative data for three physical frailty parameters in an aging, rural Indian population. Health Sci Rep. 2022;5(2):e567. 10.1002/hsr2.567 35356805 PMC8938919

[51] Stolz E , Mayerl H, Godin J, et al. Reliability of the frailty index among community-dwelling older adults. J Gerontol A Biol Sci Med Sci. Published online September 20, 2023;79:glad227. 10.1093/gerona/glad227. PMC10809054 37738215

