# Supplementary material for: Longitudinal Quantiles of Frailty Trajectories Considering Death: New Insights into Sex and Cohort Differences in the Reference Curves for Frailty Progression of Older European
Source: J Gerontol A Biol Sci Med Sci. 2024 Feb 23;79(4):glae060. doi: 10.1093/gerona/glae060 (PMC10965030; doi:10.1093/gerona/glae060)
Supplement: glae060_suppl_Supplementary_Tables_S1-S2 [file glae060_suppl_supplementary_tables_s1-s2.docx]

Table S1 Items included in the Frailty Index (FI) using SHARE data

| Variable | Cut-off points |
| --- | --- |
| 1. Self-perceived health | poor = 1, fair = 0.75, good = 0.5,  very good = 0.25, excellent = 0 |
| 1. Engagement in activities requiring moderate level of energy (e.g. gardening) | monthly or more often = 0,  hardly ever/never = 1 |
| 1. Long-term illness | no = 0, yes = 1 |
| 1. Difficulties: reaching or extending arms above shoulder | no = 0, yes = 1 |
| 1. Difficulties: climbing one flight of stairs | no = 0, yes = 1 |
| 1. Difficulties: walking 100 metres | no = 0, yes = 1 |
| 1. Difficulties: lifting or carrying weights over 5 kilos | no = 0, yes = 1 |
| 1. Difficulties: using the toilet, including getting up or down | no = 0, yes = 1 |
| 1. Difficulties: bathing or showering | no = 0, yes = 1 |
| 1. Difficulties: dressing, including shoes and socks | no = 0, yes = 1 |
| 1. Difficulties: getting in or out of bed | no = 0, yes = 1 |
| 1. Difficulties: getting up from chair | no = 0, yes = 1 |
| 1. Difficulties: walking across a room | no = 0, yes = 1 |
| 1. Difficulties: eating, cutting up food | no = 0, yes = 1 |
| 1. Difficulties: eating, cutting up food | no = 0, yes = 1 |
| 1. Difficulties: doing work around the house or garden | no = 0, yes = 1 |
| 1. Difficulties: preparing a hot meal | no = 0, yes = 1 |
| 1. Difficulties: taking medications | no = 0, yes = 1 |
| 1. Difficulties: managing money | no = 0, yes = 1 |
| 1. Difficulties: using telephone | no = 0, yes = 1 |
| 1. Doctor told you had: heart attack | no = 0, yes = 1 |
| 1. Doctor told you had: high blood pressure or hypertension | no = 0, yes = 1 |
| 1. Doctor told you had: stroke | no = 0, yes = 1 |
| 1. Doctor told you had: cancer | no = 0, yes = 1 |
| 1. Doctor told you had: diabetes or high blood sugar | no = 0, yes = 1 |
| 1. Doctor told you had: chronic lung disease | no = 0, yes = 1 |
| 1. Doctor told you had: arthritis | no = 0, yes = 1 |
| 1. Doctor told you had: hip fracture or femoral fracture | no = 0, yes = 1 |
| 1. Doctor told you had: Parkinson’s disease | no = 0, yes = 1 |
| 1. Impaired orientation (date, month, year, day of week) | good = 0, < good = 1 |
| 1. Reduced appetite | no = 0, yes = 1 |
| 1. Experienced fatigue | no = 0, yes = 1 |
| 1. Bothered by: falling down | no = 0, yes = 1 |
| 1. Bothered by: fear of falling down | no = 0, yes = 1 |
| 1. Bothered by: dizziness, faints or blackouts | no = 0, yes = 1 |
| 1. Impaired vision: distance (with glasses/lenses) | excellent – good = 0, fair & poor = 1 |
| 1. Impaired vision: closeness (with glasses/lenses) | excellent – good = 0, fair & poor = 1 |
| 1. Impaired hearing | excellent – good = 0, fair & poor = 1 |
| 1. BMI deficit | BMI ≥ 18.5, BMI < 25 = 0  BMI ≥ 25, BMI ≤ 30 = 0.5  BMI < 18.5, BMI > 30 = 1 |
| 1. Grip strength (GS) in kg (by BMI) | Men:  - BMI ≤ 24, GS ≤ 29  - BMI > 24 and ≤ 28, GS ≤ 30  - BMI > 28, GS ≤ 32  Women:  - BMI ≤ 23, GS ≤ 17  - BMI > 23 and ≤ 26, GS ≤ 17.3  - BMI > 26 and ≤ 29, GS ≤ 18  - BMI > 29, GS ≤ 21 |

Note: Based on Stolz and colleagues (2016)

Table S2 Estimates of the quantile regressions with weights including education. Outcome variable FI (range=0-1).

|  |  | $\hat{\beta}_{v}^{0.1}$  $\left( SE \right)$ | $\hat{\beta}_{v}^{0.5}$  $\left( SE \right)$ | $\hat{\beta}_{v}^{0.9}$  $\left( SE \right)$ | $\tau$=0.1 vs 0.5 p-value ^a^ | $\tau$=0.5 vs 0.9 p-value ^b^ |
| --- | --- | --- | --- | --- | --- | --- |
| Quantile Regression weighted - Intermittent missing data and death | | | | | | |
| Sex | $\hat{\beta}_{s}^{\tau}$ | -0.0068* (0.0015) | 0.0025 (0.0027) | 0.0174* (0.0083) | 0.000 | 0.052 |
| Educ | $\hat{\beta}_{e}^{\tau}$ | -0.0006* (0.0002) | -0.0012* (0.0003) | -0.006* (0.001) | 0.021 | 0.000 |
| BAge | $\hat{\beta}_{\mathrm{ba}}^{\tau}$ | -0.0027* (0.0003) | -0.0073* (0.0005) | -0.0059* (0.0016) | 0.000 | 0.351 |
| Boom | $\hat{\beta}_{\mathrm{bo}}^{\tau}$ | 0.0021* (0.0002) | 0.0066* (0.0004) | 0.0244* (0.0013) | 0.000 | 0.000 |
| Age | $\hat{\beta}_{a}^{\tau}$ | 0.0012* (0.0002) | 0.0022* (0.0003) | -0.0016* (0.0006) | 0.001 | 0.000 |
| Age.Sex | $\hat{\beta}_{\mathrm{as}}^{\tau}$ | -0.0001* (0.0001) | -0.0003* (0.0001) | -0.0002* (0.0001) | 0.000 | 0.061 |
| Age.Educ | $\hat{\beta}_{\mathrm{ae}}^{\tau}$ | 0.0001* (0.0001) | 0.0004* (0.0001) | -0.0001 (0.0001) | 0.000 | 0.000 |
| Age.BAge | $\hat{\beta}_{\mathrm{aba}}^{\tau}$ | -0.0068* (0.0015) | 0.0025 (0.0027) | 0.0174* (0.0083) | 0.000 | 0.052 |
| Quantile Regression unweighted | | | | | | |
| Sex | $\hat{\beta}_{s}^{\tau\mathrm{UW}}$ | -0.0071* (0.0014) | 0.0010 (0.0021) | 0.0354* (0.0053) | 0.000 | 0.000 |
| Educ | $\hat{\beta}_{e}^{\tau\mathrm{UW}}$ | -0.0007* (0.0002) | -0.0014* (0.0003) | -0.0062* (0.0005) | 0.002 | 0.000 |
| BAge | $\hat{\beta}_{\mathrm{ba}}^{\tau\mathrm{UW}}$ | -0.0012* (0.0002) | -0.0034* (0.0003) | -0.0030* (0.0008) | 0.000 | 0.618 |
| Boom | $\hat{\beta}_{\mathrm{bo}}^{\tau\mathrm{UW}}$ | 0.0010* (0.0002) | 0.0031* (0.0002) | 0.0115* (0.0005) | 0.000 | 0.000 |
| Age | $\hat{\beta}_{a}^{\tau\mathrm{UW}}$ | 0.0012* (0.0001) | 0.0024* (0.0002) | 0.0008 (0.0005) | 0.000 | 0.000 |
| Age.Sex | $\hat{\beta}_{\mathrm{as}}^{\tau\mathrm{UW}}$ | -0.0001* (0.0001) | -0.0002* (0.0001) | -0.0003* (0.0001) | 0.000 | 0.071 |
| Age.Educ | $\hat{\beta}_{\mathrm{ae}}^{\tau\mathrm{UW}}$ | 0.0001* (0.0001) | 0.0003* (0.0001) | 0.0002* (0.0001) | 0.000 | 0.270 |
| Age.BAge | $\hat{\beta}_{\mathrm{aba}}^{\tau\mathrm{UW}}$ | -0.0071* (0.0014) | 0.0010 (0.0021) | 0.0354* (0.0053) | 0.000 | 0.000 |

Notes: Robust standard errors in parenthesis. Age centred at 65 years old. Educ: Education (in years centred at mean 9.6 years). BAge: Baseline age (in years centred at 65 years old). Boom: indicator, 1= born in 1946 or later, 0= born before 1946. Interactions of variables are symbolised with a dot. ^a^ p-value for H_0_: $\beta_{v}^{0.1}=\beta_{v}^{0.5}$; ^b^ p-value for H_0_: $\beta_{v}^{0.5}=\beta_{v}^{0.9}$; * if p<0.05 for H_0_: $\beta_{v}^{\tau}=0$.
